# AI chatbots and (mis)information in public health: impact on vulnerable communities

**DOI:** 10.3389/fpubh.2023.1226776

**Published:** 2023-10-31

**Authors:** Dan W. Meyrowitsch, Andreas K. Jensen, Jane B. Sørensen, Tibor V. Varga

**Affiliations:** ^1^Global Health Section, Department of Public Health, University of Copenhagen, Copenhagen, Denmark; ^2^Section of Biostatistics, Department of Public Health, University of Copenhagen, Copenhagen, Denmark; ^3^Section of Epidemiology, Department of Public Health, University of Copenhagen, Copenhagen, Denmark

**Keywords:** LLM, chatbot, public health, vulnerable communities, Global South, ChatGPT, Artificial Intelligence

## Introduction

Artificial Intelligence (AI)-based chatbots are considered one of the most innovative digital advancements in recent times. The public release of the AI chatbot ChatGPT (GPT-3.5) by OpenAI in November 2022 attracted massive attention with more than 100 million monthly active users. GPT-4 was released in March 2023 and is presently available for paid subscribers.

## ChatGPT and public health

ChatGPT is a Large Language Model (LLM)-based pre-trained model. The formal announcement from OpenAI indicates that ChatGPT's training period for v.3.5 ended in December 2021. Competing chatbots are under development by Google and Meta, and probably most relevant to the general biomedical research community, BioGPT, trained on tokens from NCBI's PubMed, has recently been released (1). An integration of LLM-based chatbot functions in an internet browser has already been launched.

Chatbots will advance in ways beyond our present imagination, and they carry a huge potential for democratizing knowledge. Their potential extends to the crucial task of reducing inequalities in access to evidence-based information relevant to health promotion by facilitating equitable access to health-related information. This is particularly relevant in addressing health disparities between the Global North and the Global South and for marginalized populations within and across nations. Another potential benefit of chatbot-facilitated health information is the option for users to choose a relevant language. However, we also see potential risks. Generally, ChatGPT is perceived and used as an advanced search engine that can generate detailed and elaborate answers through a real-time dialog with the user. As the underlying machine learning methods are not well-positioned to distinguish between factually correct and incorrect information (2), ChatGPT regularly makes factual mistakes and provides imprecise information, called “hallucinations” (3). In individual users' sessions, it is possible to correct and influence answers related to health-related questions. If users share counterarguments and refer to peer-reviewed scientific articles, links to webpages, or even present non-sensical argumentation, ChatGPT will excuse its previous error and emphasize that the information promoted by the user is correct. Subsequently, if the user inquiries about the same question again in the same session, ChatGPT will answer by reproducing the new information.

As ChatGPT is based on an LMM architecture, it has two levels of memory. One is the short-term memory that defines the context window, which is the amount of preceding text that it uses to generate a response in each user session. It is this sliding – but limited – context window that gives the user the impression that it is possible to teach the bot new information in real time. This context is, however, neither shared between different sessions for the same user nor between different users' sessions. The chatbot's long-term memory, on the other hand, is the result of the bot being trained on a large corpus of text. OpenAI has intentionally not released the technical details of training ChatGPT due to the competitive nature of the AI landscape, but it is known that ChatGPT has been fine-tuned using Reinforcement Learning from Human Feedback (RLHF) to improve the validity of its responses (4). Even though ChatGPT uses an open-source technology (the underlying software is accessible to the public), it is difficult to ascertain how ChatGPT develops and improves the model, and especially to which degree data from the user sessions enter into the corpus and thus become part of the bot's long-term memory. According to OpenAI's privacy policy (5), they collect personal information such as user input to improve their services, conduct research, and develop new programs and services. As of April 25, 2023, OpenAI introduced the ability to turn off the chat history in ChatGPT and specifically stated that when the chat history is disabled, conversation histories will not be used to train and improve the underlying model (6). This suggests that without some kind of targeted filtering or active human intervention, incorrect information supplied in one or more chat sessions could at some point enter the training corpus and thus, over time, become part of the bot's long-term memory.

In the lack of access to valid information regarding the training of ChatGPT, we decided to ask ChatGPT about the influence of its users on its own dissemination of health-related information. We varied our questions and asked in different ways using a wide range of grammar and rhetorical approaches. No matter our approach, ChatGPT always replied that information corrected by a user will affect its response to other users with the same question.

## Concerns regarding AI-powered chatbots

Due to the lack of transparency regarding the development of the model, we express concern over the possibility that groups of users may select specific health topics and influence ChatGPT and similar AI technologies to propagate false health-related information, a phenomenon that is already widespread, e.g., through the use of social media (7–9). In contrast to existing internet-based mis- and disinformation, chatbots can disseminate incorrect or biased healthcare information in a way that will be difficult to see through in terms of perceived quality and details. This problem is exacerbated by the observation that humans generally find AI-generated texts equally or more credible than human-written texts (10). Thus, we believe that chatbots, such as ChatGPT, will likely magnify the already existing problem of misinformation in exponential proportions and can threaten public health globally. However, it is important to note that as of 2023, there remains a knowledge gap in accurately assessing the potential extent to which chatbots like ChatGPT might amplify the problem of healthcare-related misinformation and disinformation, given the complex nature of social dynamics that demand detailed modeling of network structures and interactions (8). As a worst-case, despite efforts to limit such scenarios (11, 12), deliberate manipulation of chatbots (e.g., by economic and political interest groups, cybercriminals, or “disinformation farms”) can be used to harm states, communities, and health services (13, 14). The extent of safeguards and personnel dedicated to countering such risks remains unclear. Thus, transparency in assessing the potential scale and risks of organized manipulation efforts is crucial to comprehend their impact on AI chatbots. We believe developers of AI chatbots should make reports on their monitoring capabilities, vulnerabilities, and vigilance systems publicly available so that the public is sufficiently informed about their systems' resilience against misinformation and disinformation threats. The current inability of chatbots to distinguish varying levels of evidence-based knowledge presents a pressing challenge for global public health promotion and disease prevention. Importantly, chatbots could potentially exacerbate the existing health inequality between the Global South and the Global North.

## Recommendations and conclusions

We strongly encourage individuals and companies who engage in the further development and implementation of AI-powered chatbots and similar technologies to take their responsibility as gatekeepers seriously. To address these concerns, we propose a multi-faceted approach. First, we suggest enhancing content validation by establishing partnerships with advisory boards, health organizations, and fact-checking entities to strengthen the accuracy and reliability of the health information disseminated. Second, we advocate for comprehensive user education initiatives through collaboration with governments, educational institutions, and tech companies (15). In our opinion, these initiatives would empower individuals to critically evaluate information provided by chatbots and recognize their limitations, although this will need to be rigorously evaluated by research as others have also proposed (9). Digital literacy and the ability to identify reliable health sources should be core components of these programs (16). Third, continuous investment in the research and development in refining AI algorithms is crucial to reduce factual errors and ‘hallucinations'. Here, the ultimate goal is to enable chatbots to better differentiate accurate health information from misinformation; ironically, AI solutions could be helpful in the prevention of AI misinformation on a massive scale. Fourth, we emphasize the importance of transparency standards in AI model development. This includes providing detailed insights into training processes, sources, datasets and tokens used, and applied quality control measures. Furthermore, we advocate for the establishment and enforcement of legal frameworks that hold companies accountable for the potential harm caused by their AI products (16). Last, we believe it is imperative to promote AI technologies that benefit all communities, regardless of their geographical or economic status. Special attention should be given to addressing the unique challenges faced by vulnerable populations in the Global South and those that are most susceptible to inaccurate health information (15). Bridging health information disparities is paramount. To implement this recommendation, we propose the establishment of diverse advisory panels responsible for assessing the development and performance of AI chatbots through an “equality lens” (17). These panels would work to establish benchmarking frameworks that ensure AI chatbots contribute to fostering fairness and inclusivity in healthcare information dissemination.

By implementing these recommendations, stakeholders can take proactive steps to mitigate the risks associated with AI chatbots and leverage their potential to advance global public health. This approach aims to prevent crises similar to the spread of conspiracy theories and misinformation observed, for example, during the COVID-19 pandemic (18–20), ultimately safeguarding public health worldwide.

## Author contributions

All authors listed have made a substantial, direct, and intellectual contribution to the work and approved it for publication.
